# Emerging Thawing Technologies for Frozen Muscle Foods: Mechanisms, Quality Impacts, and Industrial Prospects

**DOI:** 10.3390/foods15111991

**Published:** 2026-06-03

**Authors:** Yaping Wang, Yantong Liang, Yanyan Huang, Lang-Hong Wang, Qinglin Sheng, Nana Zhang

**Affiliations:** 1 College of Food Science and Technology, Research Center of Food Safety Risk Assessment and Control, Northwest University, Xi’an 710069, China; wyping1023@163.com; 2 Guangdong Provincial Key Laboratory of Intelligent Food Manufacturing, School of Food Science and Engineering, Foshan University, Foshan 528225, China; ytongleung@163.com (Y.L.); huang_yanyan@fosu.edu.cn (Y.H.); wlhong@fosu.edu.cn (L.-H.W.); 3 School of Food Science and Technology, Jiangnan University, Wuxi 214122, China

**Keywords:** muscle foods, thawing technologies, physical fields, physicochemical quality, drip loss, lipid oxidation

## Abstract

Freezing is an important technique for preserving muscle foods (encompassing mammalian meat, poultry, and seafood). However, traditional thawing methods have several drawbacks, including excessive drip loss, nutrient leaching, and overall quality degradation. To address these issues, emerging technologies such as high-voltage electric field, ohmic, microwave, ultrasound-assisted, low-temperature combined with high-humidity (LHT), radiofrequency (RF), and vacuum thawing have been developed. Despite their potential, existing literature frequently focuses on standalone methods or isolated engineering parameters, leaving a critical knowledge gap regarding their comparative industrial viability and combined synergistic effects. Based on a comprehensive literature search across major scientific databases, the changes in meat product quality during the thawing process were systematically discussed, followed by an exploration of the principles and applications of these innovative methods. Crucially, comparative findings indicate that LHT thawing most effectively preserves water-holding capacity (WHC) and minimizes lipid oxidation. In contrast, RF thawing provides the optimal balance between rapid thawing rates and uniform quality retention for large-scale operations, while hybrid approaches (e.g., microwave combined with ultrasound) successfully balance high-speed processing with the prevention of structural degradation. Furthermore, the practical applications of these technologies in the food industry were presented, emphasizing the growing trend of combining multiple techniques. The advantages and disadvantages of the thawing process are analyzed to provide theoretical references and practical insights for enhancing the quality of commercial meat products.

## 1. Introduction

With global population growth and economic development, the meat industry has experienced unprecedented expansion. To meet international demand, vast quantities of various meats and seafood, primarily beef, pork, poultry, and fish, are processed and transported globally through complex cold chain logistics. Typically, freezing at −18 °C or lower remains the most critical and widely adopted technique to ensure the freshness, microbiological safety, and extended shelf life of these products during long-distance global trade [1]. However, the immense scale of the global frozen meat trade brings about significant economic challenges. The energy consumption associated with prolonged freezing and cold storage, coupled with the inevitable requirement of thawing before secondary processing or consumption, imposes a tremendous economic burden on the industry [2]. Furthermore, traditional thawing methods often result in considerable drip loss, which directly translates to a reduction in sellable weight and substantial financial deficits worldwide. Therefore, the implementation of innovative thawing technologies is vital not only for preserving quality but also for reducing energy-consuming thawing times and minimizing weight-loss-related costs, thereby enhancing the overall profitability and sustainability of the global meat industry [3]. In this review, to provide a comprehensive overview of thawing technologies, the discussed food matrices encompass not only mammalian and poultry meat but also seafood and aquatic muscle foods (such as fish and crustaceans), collectively referred to as “muscle foods” where appropriate.

The methods and procedures used to thaw muscle foods significantly impact their water retention, lipid stability, and protein integrity. During the thawing phase, increased drip loss leads to the depletion of essential nutrients, including soluble vitamins, proteins, and lipids [4]. Traditional thawing methods, such as water and air thawing, are not only time-consuming but also exacerbate this drip loss. This can cause secondary structural damage to the muscle foods and create a favorable environment for microbial proliferation. Consequently, various bacteria can thrive, leading to product deterioration and posing potential food safety risks, which ultimately result in economic losses for food enterprises. To meet the growing consumer demand for high-quality meat products, the meatpacking and broader food industries are in urgent need of advanced thawing solutions.

These emerging technologies aim to maintain the desirable quality characteristics of frozen muscle foods during thawing while simultaneously enhancing the economic efficiency of the food industry. Consequently, a rapidly increasing number of novel thawing techniques are being developed [5,6,7,8]. As summarized in Table 1, these modern approaches are applied across various types of muscle foods, each offering distinct advantages. For instance, microwave thawing offers rapid processing speeds; radio-frequency thawing provides deeper and more uniform volumetric heating; ultrasonic thawing enhances heat transfer via cavitation; vacuum thawing effectively prevents protein and lipid oxidation; high-voltage electric field thawing assists in inhibiting microbial growth; ohmic thawing mitigates nutrient degradation; and low-temperature, high-humidity thawing reduces lipid oxidation while improving protein solubility [7,9,10,11,12].

However, several existing reviews on this topic often suffer from significant limitations, as they predominantly focus on standalone technologies or isolated engineering parameters, failing to provide a holistic, multi-species comparison of combined physical fields on comprehensive quality attributes [28,29]. Therefore, the primary objective of this review is to critically evaluate emerging thawing technologies and their efficacy in mitigating quality deterioration in muscle foods compared to traditional methods. The specific objectives of this review are: (i) to compare the mechanistic differences between novel and conventional thawing processes; (ii) to evaluate the impact of these emerging technologies on the physicochemical, microbiological, and structural properties of different muscle matrices; and (iii) to identify the major technical and economic obstacles preventing their large-scale industrial implementation.

Ultimately, presenting these novel thawing technologies aims to answer a critical question for the food industry. To address this, this review establishes a unified framework driven by quality attributes (physicochemical, nutritional, and structural) and industrial viability. The overarching goal is to bridge the gap between laboratory-scale academic research and commercial scalability, serving as a high-value reference for both researchers and enterprises to successfully implement these technologies in the global muscle food sector.

## 2. Literature Search and Review Framework

To systematically address the aforementioned research questions, a literature search was conducted using major scientific databases, including Web of Science, Scopus, and PubMed. Keywords included combinations of (“thawing” OR “thawing”) AND (“meat” OR “seafood” OR “muscle foods”) AND (“novel technology” OR “high-voltage electric field” OR “ohmic” OR “microwave” OR “radiofrequency” OR “ultrasound” OR “vacuum”). The inclusion criteria focused on peer-reviewed research articles and authoritative reviews published primarily between 2010 and 2026.

To avoid a merely descriptive compilation, the discussion of each thawing technology is organized around a consistent comparative framework. The evaluation criteria prioritized across all technologies include: (i) operational efficiency (thawing rate and temperature uniformity); (ii) product quality indices (water-holding capacity, protein oxidation/denaturation, and lipid oxidation); (iii) microbiological safety; and (iv) industrial viability (energy demand, scalability, and equipment readiness). Following this methodological framework, the structure of the manuscript is organized as follows: first, the changes in meat product quality during the thawing process were discussed. Second, the underlying principles, operational efficiencies, and specific applications of various novel thawing technologies were explored. Finally, the practical applications of these technologies in the food industry were presented, along with a critical synthesis and future perspectives regarding their industrial viability and scalability.

## 3. Changes of Quality Characteristics of Meat Products During Thawing

Before detailing the specific quality changes during thawing, it is crucial to recognize that the transferability of thawing mechanisms and quality endpoints depends heavily on the specific biological matrix of the muscle food. Mammalian and poultry meats exhibit significant physiological and biochemical differences compared to seafood and aquatic muscle foods. For instance, fish and crustaceans possess substantially lower collagen content and structurally weaker connective tissues than mammalian meat, making their myocommata and myofibrillar structures highly susceptible to severe mechanical disruption from ice crystal formation and melting during thawing [30,31]. Furthermore, the lipid composition of aquatic muscle foods is distinctively rich in highly reactive polyunsaturated fatty acids (PUFAs). This composition makes seafood significantly more vulnerable to rapid lipid oxidation and the development of off-flavors (e.g., fishy odors) during the thawing phase, especially when exposed to oxygen or external energy fields (like high-voltage electric fields or microwaves) [32]. Conversely, mammalian meats, with their robust connective tissue networks and different fatty acid profiles, generally exhibit different postmortem biochemical behaviors and color stability issues (such as myoglobin oxidation) during thawing [33,34,35]. Therefore, while novel thawing technologies demonstrate general efficacy across various species, the specific processing parameters must be strictly tailored to the inherent structural and chemical characteristics of each muscle matrix.

### 3.1. Water Holding Capacity Decreases

The ability of meat products to preserve their water under the influence of an external force is referred to as “water holding” capacity. It can be assessed using a variety of measures, including the rate at which food is lost during heating, thawing, and dripping. Depending on the mobility and location of water in the muscle, it can be generally categorized into bound water, immobilized water, and free water. While a lower proportion of free water is often associated with better water retention [36], this relationship is highly complex and not a simple one-to-one correlation. In reality, water-holding capacity depends heavily on multiple interconnected factors, including protein structure, pH, ionic strength, myofibrillar integrity, the extent of protein denaturation and oxidation, and the microstructural compartmentalization of water within the muscle. By analyzing the relative proportions of water populations within muscle foods, specifically bound water, immobilized water, and free water, it is evident that the content of immobilized water decreases while the content of free water increases following thawing [37]. Cai et al. (2018) investigated the thawing of red snapper and found that it encourages water migration [8]. It is due to the extracellular fluid turning into ice crystals during the frozen storage period, resulting in an osmotic pressure difference inside and outside the cells and the outflow of intracellular water [38]. The mechanism of water migration differs slightly among species; specifically, the inherently weaker connective tissue in aquatic muscle foods makes their extracellular matrix more susceptible to irreversible expansion by ice crystals compared to the denser perimysium found in mammalian meats like beef or pork. Consequently, the breakdown of myofibrillar protein’s structure and exposure of the hydrophobic amino acids within the protein directly causes protein reduction, water binding and migration, and water holding capacity. It is responsible for the significant decline in meat and meat product quality after thawing [39]. Maintaining the water retention of meat products during thawing has traditionally been a focus in thawing technology because if it decreases, the cell contents, including certain soluble nutrients, are lost as drip and significantly lower the nutritional value. Additionally, this nutrient-rich drip can accelerate meat deterioration by promoting bacterial growth and producing microbial pollutants throughout the product [40].

### 3.2. Protein Denaturation and Oxidation

About 20% of muscle comprises protein, which can be classified into three types based on their solubility in salt solutions and protein composition. These are conventionally classified into connective tissue (stromal) proteins, myofibrillar proteins, and sarcoplasmic proteins based on their solubility. The ability of muscle foods to retain water is primarily determined by myofibrillar proteins, tenderness is largely influenced by connective tissue (stromal) proteins, and meat color, which a crucial attribute for consumer acceptance is specifically governed by myoglobin, a specific hemoprotein within the sarcoplasmic fraction [41]. This result may be related to pigment loss and myoglobin oxidation during thawing. Meat color is primarily determined by the chemical state of myoglobin, which exists in three main forms: deoxymyoglobin, oxymyoglobin, and metmyoglobin. During the thawing process, myoglobin undergoes oxidative transformations, accelerating the conversion of bright-red oxymyoglobin and purple-red deoxymyoglobin into brown metmyoglobin, which negatively impacts the visual appeal of the meat [42].

**Figure 1:**
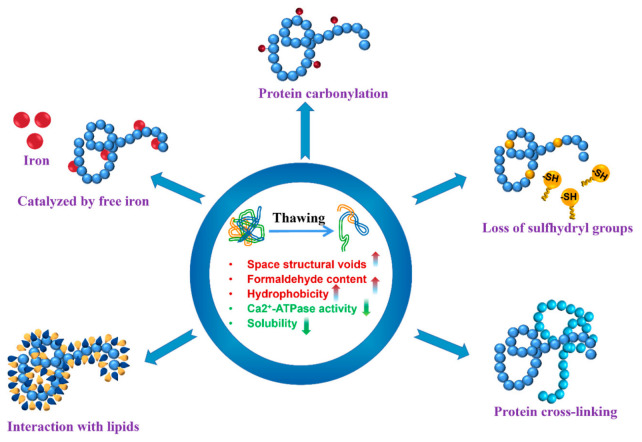
Mechanisms of protein denaturation and oxidation during the thawing of muscle foods. The central core illustrates the primary physicochemical changes induced by protein unfolding, including increased structural voids, formaldehyde content, and surface hydrophobicity, alongside decreased Ca^2+^-ATPase activity and protein solubility. The outward arrows indicate five specific biochemical pathways driving this degradation: (1) protein carbonylation, (2) loss of sulfhydryl (-SH) groups, (3) protein cross-linking, (4) lipid–protein interactions, and (5) catalysis by free iron.

The beef was thawed at 4 °C after 7 days of frozen storage and showed a decrease in proteolysis, α-helix, ionic, and hydrogen bonds, but an increase in surface hydrophobicity [13]. Similar findings were found for pork subjected to the same treatment, with thawed pork showing dityrosine, fluorescence emission wavelength, and ultraviolet second derivative spectra increased but α-helix decreased [43]. In turkeys, a decrease in Ca^2+^-ATPase activity and total sulfhydryl content was found after three weeks of freezing [44]. All these experimental results show that myofibrillar proteins and myosin are more likely to freeze and thaw throughout the thawing process. Furthermore, the thermal and freeze–thaw stability of these myofibrillar proteins is highly species-dependent. For example, fish myosin is generally less stable and denatures more rapidly at lower temperatures during thawing than mammalian myosin, necessitating stricter temperature control when thawing seafood. In general, covalent forces like disulfide bonds and non-covalent forces like hydrogen bonds, hydrophobic interactions, electrostatic interactions, and van der Waals forces contribute to the stability of protein structure. In contrast, thawing disrupts intermolecular interactions within myofibrillar proteins, such as reduced hydrogen and ionic bonding, decreased α-helix and increased β-sheet content. Protein oxidation and denaturation have a close relationship (As shown in Figure 1). With the extension of frozen storage time, the overall quality of muscle foods significantly deteriorates, as evidenced by increased myofibrillar protein denaturation and enhanced water migration [45]. Ice crystallization during freezing results in mechanical stress, which loosens and destabilizes the protein structure. When protein molecules are stretched and unfolded during the thawing process, the spatial structure of the protein is destroyed. Proteins form cross-links with each other or expose hydrophobic amino groups on the surface [46]. It is because there is an increase in the generation of reactive oxygen radicals, which cause protein oxidation to ruin the cell structure. It causes the sulfhydryl group to be converted into disulfide bonds in the protein, lowering its sulfhydryl concentration [47]. At the same time, the interaction between the catalysis of iron ions and lipid oxidation will accelerate the oxidation of proteins [48]. Notably, calpain, which is activated during the thawing process, can catalyze the degradation of myofibrillar proteins, resulting in excessive softening or a mushy texture, which negatively affects the quality [49].

### 3.3. Degree of Oxidation and Decomposition of Lipids

One of the primary causes of the decline in meat quality is lipid oxidation, which affects the flavor, texture, and nutritional value of meat and its shelf life [50]. This degradation pathway is particularly aggressive in aquatic muscle foods due to their high proportion of polyunsaturated fatty acids (PUFAs), making them significantly more vulnerable to rapid lipid hydrolysis and the generation of secondary oxidative off flavors than mammalian meats, which predominantly contain saturated and monounsaturated fats. During the thawing process, the structural disruption of cellular compartments accelerates lipid hydrolysis, leading to the breakdown of triglycerides and membrane phospholipids into free fatty acids. These accumulated free fatty acids subsequently serve as highly reactive substrates for further oxidative degradation [6]. Numerous investigations into the mechanisms of lipid oxidation in muscle foods have reported a significant increase in the concentration of thiobarbituric acid reactive substances (TBARS) during the thawing process [51,52,53]. During frozen storage, cellular water is immobilized as ice crystals, effectively halting various biochemical reactions that require an aqueous medium. Upon thawing, the melting of ice crystals re-initiates lipid hydrolysis. Concurrently, pro-oxidant substances (such as heme iron) are mobilized by the outflow of drip. This phenomenon, coupled with the relative increase in lipid concentration due to moisture loss, accelerates the overall rate of lipid oxidation and decomposition [48]. In the thawing temperature range of 0 °C to 4 °C, psychrotrophic spoilage bacteria such as *Pseudomonas fragi*, *Pseudomonas fluorescens*, *Brochothrix thermosphacta*, and *Lactobacillus* species can rapidly proliferate. These microorganisms produce enzymes that catalyze the hydrolysis of triglycerides and phospholipids, eventually increasing the accumulation of free fatty acids. The phosphoric acid in the cell membrane plays a crucial role. The freezing and thawing process destroys the integrity of the cell membrane, increases the contact between air and lipids, and promotes the oxidation and decomposition of lipids [54].

### 3.4. Microstructural Analysis

**Figure 2:**
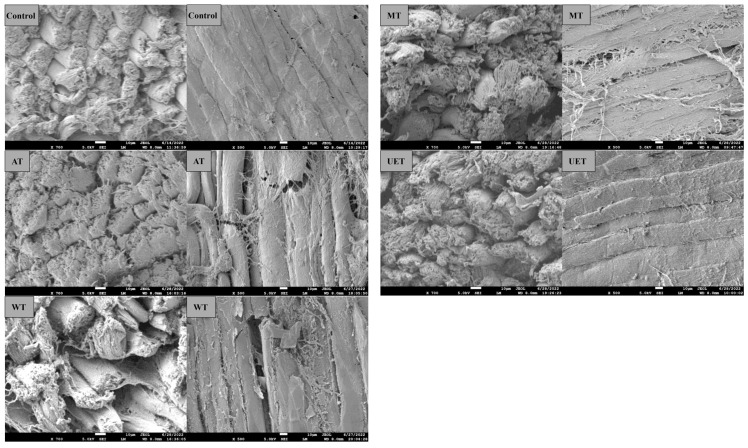
Scanning electron microscopy (SEM) images demonstrating the effects of different thawing techniques on the microstructural integrity of mutton (Reproduced with permission from Kong et al. [59], Copyright 2023). The fresh mutton control exhibits intact, tightly packed muscle fiber bundles with distinct, regular myofibrillar structures. In contrast, traditional methods such as air thawing (AT) and water thawing (WT) result in noticeable structural degradation, characterized by widened extracellular gaps, disrupted connective tissues, and torn muscle fibers caused by the melting of large, irregular ice crystals. Microwave thawing (MT) exhibits severe structural deformation, irregular fiber shrinkage, and fragmentation, which are typical consequences of localized overheating and uneven thermal distribution. Conversely, the novel combined treatment of ultrasound-assisted slightly acidic electrolyzed water thawing (UET) effectively mitigates mechanical damage, maintaining a smooth, compact, and highly complete microstructural network with minimal fiber breakage that closely resembles the fresh control group.

The basic building blocks of muscle are muscle fiber, which are elongated, multinucleated fibrous cells. When comparing matrices, the microstructural damage manifests differently: mammalian meats often show tearing in the distinct endomysium and perimysium networks, whereas aquatic muscle foods frequently exhibit severe fragmentation at the myocommata junctions due to their inherently lower collagen cross-linking. The combination of muscle fiber with fatty tissue gives the meat a special textural structure [55]. The textural structure of the meat is locally irregular and macroscopically regular using scanning electron microscopy (SEM) or transmission electron microscopy (TEM) to get a better visualisation of the microscopic structure of the meat. The dark lines at the ends of the muscle segments are called Z-lines, the dark band in the middle is about 1.5 μm wide and the bright band between the A-band and the Z-lines is about 0.4 µm, called the I-band. In the centre of the A-band is a wide striated zone of approximately 0.4 µm, called the H-band [56]. The thawing process causes protein denaturation, lipid oxidation and other muscle fibre activity, which affects the microstructure of the meat product, resulting in different textures and flavours. Microstructurally, the myofibrils are stretched and the gaps between them are severely enlarged, and the fascia and endomysium are severely torn. These gaps will form water channels through which water will leak, causing further drip loss [57]. Hsieh et al. (1980) report that heating can cause aggregation and contraction of myofibrillar proteins in bovine semitendinosus muscle, with only the Z-line clearly visible after heating, and that microwave heating has less effect on the structure of the meat than steaming and roasting heating, which result in lysis of myofibrils, while partial presence of myofibrils can be observed in the microstructure after microwave heating [58]. Overall, applying these novel thawing techniques is highly beneficial for maintaining the stability of the muscle’s microstructure (as shown in Figure 2). Kong et al. compared the microstructure of lamb meat under different thawing methods and found that samples thawed by air thawing (AT) and water thawing (WT) exhibited extensive muscle fiber breakage. In the microwave thawing (MT) group, although there was no muscle fiber breakage, the surface was rough and the gaps between muscle fibers were large. Compared to other thawing groups, the microstructure of samples treated with ultrasound-assisted slightly acidic electrolyzed water thawing (UET) was more compact and intact. Furthermore, the thawing medium, in which slightly acidic electrolyzed water inhibited protein and lipid oxidation in the samples, helped to preserve their microstructure [59].

## 4. Principle and Application of Novel Thawing Technology

Driven by economic constraints, both industrial food processing facilities and domestic households frequently rely on conventional techniques, such as air and running-water thawing. The thawing temperature, thawing time, and thawing method determine the quality of frozen meat after thawing. The purpose of air thawing, which takes a long time and has significant drawbacks for some bulky meat items, is to control the temperature and flow rate of air in the environment to facilitate heat transfer with frozen products [60]. Running water thawing takes less time than air thawing, but meat products are more prone to grow bacteria during thawing, raising the danger to food safety [61]. When the water is thawed, the nutrients in the meat are also easily lost when washing with the water. With global advancements in thawing technology, some emerging thawing technologies have outstanding performance in thawing frozen meat, which can effectively alleviate the problems caused by the thawing process of frozen meat.

To systematically understand these emerging thawing technologies, they can be classified based on their underlying physical mechanisms into two primary categories: Electrical/Electromagnetic Technologies and Mechanical/Thermodynamic Technologies. The first category involves the application of electrical energy or electromagnetic waves, encompassing both non-thermal electrostatic interactions and thermal volumetric heating (which generates heat internally from within the product to the outside). Based on the frequency and nature of the applied field, this category is rigorously subdivided into: (i) High-Voltage Electric Field (HVEF) thawing, which functions primarily as a non-thermal technology utilizing stable direct-current (DC) high voltage to generate plasma and ionic wind without relying on frequency oscillations; (ii) Ohmic thawing (a thermal method), which relies on low-frequency alternating current (AC) passing directly through the conductive food matrix; and (iii) Electromagnetic wave thawing (also a thermal method), which employs high-frequency electromagnetic fields, specifically Microwave (MW) and Radiofrequency (RF) thawing, to induce rapid dipole rotation and ionic friction. The second category encompasses mechanical and thermodynamic approaches, which can be distinctly sub-labeled based on their driving forces. The mechanical sub-label includes Ultrasound-assisted thawing (utilizing mechanical acoustic waves for cavitation). In contrast, the thermodynamic sub-label includes Vacuum thawing (leveraging phase changes and latent heat at reduced pressure), and Low-temperature combined with high-humidity (LHT) thawing (relying on convective heat transfer and humidity-induced surface condensation to optimize surface thermodynamics). This standardized classification is strictly adhered to in the subsequent sections to clarify the distinct operational principles of each method. As shown in Table 1, the different thawing techniques are used in meat products.

### 4.1. High-Voltage Electric Field Thawing

**Figure 3:**
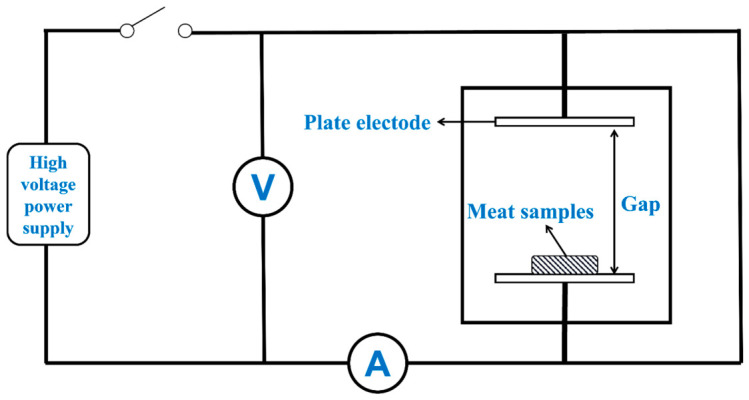
Schematic diagram of the high-voltage electric field (HVEF) thawing system. The setup features a parallel plate electrode configuration connected to a high-voltage power supply. It should be noted that this parallel plate configuration serves purely as a conceptual illustration to demonstrate the foundational sub-discharge electrostatic principle. In practice, as detailed in the reviewed literature, most experimental and industrial setups predominantly employ needle-electrode geometries. Operating strictly in the sub-discharge regime (below the air breakdown threshold), the applied electric field accelerates thawing by inducing the rapid polarization and reorientation of water dipoles within the meat, thereby disrupting ice crystal networks without generating thermal heating or corona discharge.

High-voltage electric field (HVEF) thawing is an emerging technology that, when operating strictly in the sub-discharge regime, functions as a non-thermal process to accelerate the thawing process primarily through the electrostatic enhancement of heat and mass transfer. As shown in Figure 3 the thawing of the high voltage electric field is to use electronic lines to generate stable, continuous and controllable direct-current high voltage, forming an artificial integrated effect field, further changing the transfer of heat and quality in the field [62]. As early as the 1990s, HVEF technology was widely used in food processing. HVEF thawing was initially used to sterilize food, which was crucial in preventing and eradicating bacteria. It is one of the low-temperature sterilizing technologies with the greatest promise. The in-depth study of HVEF thawing shows that HVEF thawing performs well in assisting food thawing [7]. Mechanistically, it is crucial to distinguish between two different physical regimes within HVEF applications. At sub-discharge field strengths, HVEF operates primarily through electrostatic effects on water dipoles and ionic transport, facilitating the reorientation of water molecules and accelerating ice melting without generating plasma. However, when the applied field strength exceeds the air breakdown threshold, which often dictated by specific electrode configurations (such as needle tips), narrow gap distances, and excessive voltage, the process transitions into a corona discharge regime. In this distinct regime, cold plasma is generated close to the electrode, producing highly energetic ion winds. When these charged ion wind particles interact with the surface of the muscle foods, they transfer kinetic energy to the ice crystals, further accelerating heat conduction [63]. Consequently, while the sub-discharge regime remains strictly non-thermal, the corona discharge regime introduces a localized, micro-thermal and convective contribution at the food–electrode interface due to this kinetic energy transfer. The impact of HVEF treatment with various needle electrodes on beef was investigated to enhance the thawing process of high voltage electric field. It was found that the water-holding capacity, solubility and gel strength of beef myofibrillar protein increased first and then decreased with the number of electrodes [13]. A similar experiment was conducted on chicken, and it was found that the thawing time of frozen chicken breast could be shortened by increasing voltage and decreasing the electrode gap. Under a 3 kV/cm electric field strength, the thawing time was reduced to approximately 43% of the time required under natural conditions. Another intriguing conclusion from this experiment is that the purge (cooking loss) of chicken would first increase with an increase in voltage before abruptly decreasing, which offers theoretical justification for choosing the best thawing voltage [14]. High-voltage electric fields may quickly thaw meat, but they also use much energy and regularly change the interior temperature of the meat, which can easily result in variable meat quality after thawing [6]. HVEF are effective in reducing the microbiological and total volatile nitrogen content of thawed fish products, preventing them from becoming too fishy and inedible after thawing. In contrast to sub-discharge HVEF treatments, thawing under corona discharge conditions frequently accelerates lipid oxidation and color change [15]. This quality deterioration is directly attributable to the generation of reactive oxygen species, particularly ozone, caused by the ionization of air. It is important to emphasize that ozone generation and ion winds are not inherent to HVEF thawing itself; rather, they are specific outcomes of the corona discharge regime. Consequently, if the applied voltage and electrode geometry are not carefully optimized to stay below the breakdown threshold, the resulting ozone accumulation renders muscle foods, especially seafood rich in polyunsaturated fatty acids highly susceptible to severe lipid oxidation and protein denaturation [64].

### 4.2. Ohmic Thawing

**Figure 4:**
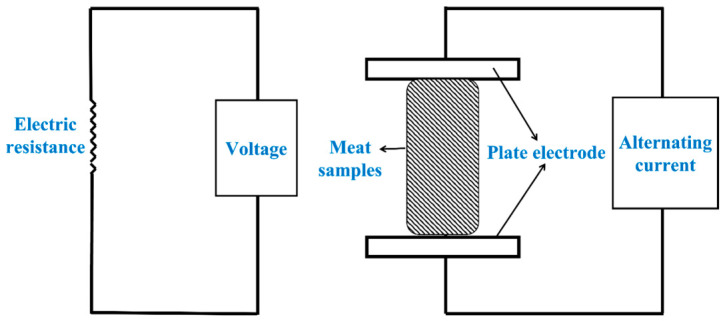
Schematic diagram of the ohmic thawing principle. In this system, the meat sample is placed directly between two plate electrodes, acting as an electrical resistor within the circuit. When an alternating current is applied, rapid and uniform volumetric heating is generated internally throughout the meat via the Joule effect, significantly accelerating the thawing process without relying on external heat transfer.

As shown in Figure 4, Ohmic thawing, a low-frequency electrical thawing technique, is a process that uses an electric current to pass through the food to achieve thawing to heat the food. Ohmic heating is a quick volumetric heating method that keeps both liquid and solid components of meat products warm simultaneously [65]. Ohmic thawing is particularly suitable for some meat products with low thermal sensitivity and low thermal conductivity, and such a thawing method can maintain the nutrient content and particle integrity of the food to a greater extent. Compared to conventional methods, ohmic heating can significantly accelerate the thawing process under appropriate parameters. For instance, in a specific study evaluating frozen tuna fish under voltage gradients of 40 to 60 V/cm, ohmic thawing increased the thawing rate by more than five times compared to conventional water immersion, while simultaneously mitigating drip loss [10]. To explore the changes in conductivity in the ohmic thawing process, an experiment was conducted to investigate the effect of parallel and series current flows on fish in the ohmic thawing circuit, and it was found that the conductivity in parallel was higher [17]. Studies have demonstrated that the electrical conductivity of meat products increases linearly with increasing temperature within a specific temperature range [66]. For instance, Cevik et al. (2021) demonstrated the robustness of ohmic thawing, finding that variations in the initial fat content of beef did not significantly affect the required thawing duration [16]. Therefore, rather than having a ‘negligible’ effect, ohmic thawing actively minimizes quality degradation, successfully preserving the nutrient content and particle integrity of the food matrix compared to traditional convective thawing methods. The ecologically friendly ohmic thawing method has numerous drawbacks. It is inappropriate for non-conductive materials when their water content is very low or dry. It is required to improve the mechanical performance and the process parameters to achieve industrial applications. The electrical conductivity of both the solid and liquid phases affects how quickly they warm up during ohmic thawing. In comparison to liquids, poor conductivity solids will exhibit thermal hysteresis at low concentrations; nevertheless, at greater concentrations, particles may heat up more quickly than fluids. This occurs because a bigger amount of the total current is forced through the particles as the solid content rises and the current route through the fluid becomes more complicated. As a result, the particles may generate energy at a faster pace, which would increase the relative heating rate [67]. Due to this potential problem, ohmic thawing is not suitable for thawing muscle foods with an excessively high moisture content or those exhibiting a massive difference in electrical conductivity before and after freezing. For example, the electrical conductivity of frozen shrimp is only one-hundredth (1/100) of that of thawed shrimp [68]. As the current flows through the thawed part of the block, the shrimp in that part may cook, while the rest of the block remains frozen. Ohmic thawing offers a homogeneous temperature distribution for products with uniform electrical conductivity compared to conventional methods. However, for heterogeneous food matrices, this method requires careful parameter control to prevent localized thermal runaway and successfully prevents microbial development. However, the direct contact between the electrodes and the product poses a potential toxicological risk due to complex electrochemical reactions at the electrode–tissue interface [69]. Specifically, the applied electrical current can induce electrode corrosion, leading to the undesirable migration of metallic ions from the electrode material directly into the muscle foods. Additionally, localized electrolysis may generate reactive byproducts that compromise food safety. Consequently, rather than being negligible, the comprehensive impact of ohmic thawing on the biochemical quality and safety of various muscle foods requires rigorous validation under diverse operational parameters.

### 4.3. Microwave Thawing

Microwave thawing is a volumetric heating method that utilizes high-frequency electromagnetic waves, typically operating within the globally allocated Industrial, Scientific, and Medical (ISM) radio bands of 915 MHz or 2450 MHz-to generate internal heat within frozen products [70]. The polar molecules in frozen meat absorb microwaves and convert the obtained electric field energy into heat for molecular motion, which raises the meat product’s internal temperature to achieve the purpose of thawing. This process occurs while the dielectric oscillates at high speed with the high-frequency alternating electromagnetic field and continuously rearranges the new molecular order [71]. The high penetrating ability of microwaves can promote the violent movement of water molecules inside the meat product to generate heat to melt the remaining ice crystals [72]. The dielectric properties of the heated material are the primary factor influencing the effect of microwave thawing. Since temperature, moisture, salt, sugar, and other substances can affect a food product’s dielectric properties, different material systems have very different microwave heating characteristics. Glass, heat-resistant polymers, and ceramics may all be penetrated by microwaves, and strong penetration and absorption can considerably increase how well microwave heat is used [73]. By using a novel thawing technique, Wang et al. (2020) sought to lessen the impacts of the thawing process on pork. They discovered that microwave thawing allowed pork to preserve its high water-holding capacity because the gelation of the myofibrillar proteins was flatter and smoother [74]. Microwave thawing is prone to local overheating, and an in-depth investigation of microwave thawing techniques revealed that microwave combined with air convection thawing showed fewer changes in water holding capacity, color, thiobarbituric acid reactive substances (TBARS) and protein solubility [18]. It was discovered that thawing meat would cause the muscle fiber gap to widen, but using microwave thawing technology may greatly reduce this occurrence and preserve the fish’s textural features to a considerable extent [75]. Similar conclusions were reached by Peng et al. (2021) when utilizing microwaves to thaw pork, and when vacuum thawing of meat was added, the thermal stability of actin and its tertiary structure increased [11]. The simultaneous thawing of the food inside and outside during microwave thawing considerably decreases the time required for thawing and makes the process more effective and economical. In the future, microwave thawing can be combined with other techniques like ultrasound, air convection, or water immersion to improve the combined microwave thawing process for meat products because uneven microwave heating can cause localized overheating of frozen foods, reducing the quality of thawed products [76].

The main issue with microwave thawing is that the energy is not dispersed evenly due to the structure of frozen meat items and the variety of water, ice, protein, fat, and sugar compounds they contain. A novel strategy to overcome the drawbacks of microwave thawing technology is to combine it with other technologies. Microwaves and magnetic nanoparticles offer optimum thermal stability and gelation characteristics. Because of the two factors working together, thawed meat displays stable secondary and tertiary protein structures [77]. In addition, the immobilized water in them relative to fresh samples has not changed much. It is still challenging to get this technology widely used in practical applications. Using a microwave and infrared light combination, Hu et al. discovered that thawing pork with both the inside and outside heated prevented quality degradation brought on by unequal interior and external heating. The two combinations increased the textural qualities of the pork while delaying the degree of lipid oxidation of the thawed meat, better maintaining the secondary structure of the proteins, and thawing meat at a slower rate than microwave thawing [78].

In recent years, to overcome the problems of uneven heating and localized overheating caused by single microwave thawing, combining microwaves with other physical fields has become a key research focus in this area. For example, recent studies have shown that integrating microwave energy with ultrasound technology can significantly stabilize the secondary and tertiary structures of proteins in meat products, minimizing protein denaturation and water migration while preserving the muscle fiber architecture [79]. The cavitation bubbles generated by ultrasound can themselves assist in thawing by raising the temperature of frozen foods through mechanical and thermal effects [80], and their synergistic application with microwave systems further improves the overall thawing efficiency and meat quality retention. In the future, technological breakthroughs in the meat processing industry will largely depend on deeply embedding microwave technology into hybrid multi-physics systems, and ultimately applying artificial intelligence (AI)-based model control systems to optimize these combined parameters in real time and dynamically, thereby ensuring precision, uniformity, and high quality in industrial thawing processes [81].

### 4.4. Radiofrequency Thawing

**Figure 5:**
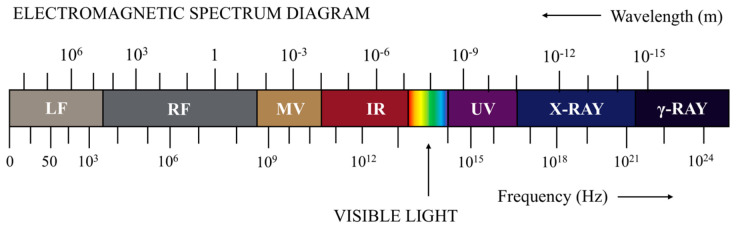
Radio frequency band distribution in electromagnetic wave spectrum. Original figure.

The principle of radiofrequency (RF) thawing is that the internal ions of meat products move in the opposite direction of charge in the high-frequency electromagnetic field, resulting in friction and vibration between ions and dipole rotation to generate heat inside the product, so as to achieve rapid thawing of the product. Compared to microwave technology, RF thawing operates at lower frequencies typically within the allocated ISM bands of 13.56, 27.12, and 40.68 MHz (falling within the broader 10 to 300 MHz range, Figure 5). This corresponds to free-space wavelengths of approximately 1 m to 30 m [82]. This longer wavelength inherently provides a significantly greater penetration depth, making RF systems highly suitable for uniform volumetric heating of large commercial frozen blocks. Palazoglu et al. (2017) also found that radiofrequency thawing was more uniform [19]. They compared radiofrequency thawing with microwave thawing in frozen shrimp samples. The results showed that the time of microwave thawing was shorter, but the local surface overheating was generated during microwave thawing, while the radiofrequency treatment had uniform temperature distribution and no local surface overheating. Chen et al. (2021) compared microwave thawing with radio-frequency thawing, and found that the higher the penetration depth of radio-frequency thawing, the higher the energy generated by the interaction between food and electromagnetic field, and the more uniform the distribution, thus helping to minimize the uncontrolled heating [21]. The characteristics of radiofrequency thawing were studied by Yang et al. (2019), and it was found that at the frequency of 27.12 MHz, the dielectric properties of fish gel increased sharply [83]. After thawing, the characteristics of fish gel could maintain the original characteristics without being affected by thawing, and the degree of lipid oxidation and protein denaturation was low.

Rf thawing technology has better uniformity than microwave thawing technology, but due to the inevitable uneven distribution of electromagnetic field in the material and cavity, there is still a problem of edge overheating. The electromagnetic field of an RF device between two parallel electrodes depends on the geometry, especially the potential of the top electrode. It is found that the frozen samples with high moisture content can be pretreated to make the electrode protrusion of the RF thawing device close to the size of the sample, so as to achieve the goal of uniform temperature distribution at the end of the sample. This indicates that the gap between electrodes affects the heating uniformity under static conditions. In addition to the gap between electrodes, the speed of the conveyor belt plays an important role in the motion condition [84]. The motion and rotation of the sample also redistribute electromagnetic fields and heat in the food, thereby improving RF heating uniformity and further optimizing RF technology. The results show that the RF thawing on the conveyor belt improves the heating uniformity slightly [20]. The future can introduce mathematical modeling methods and simulate fairly complex models of moving food in industrial scale systems, optimizing RF thawing technology.

### 4.5. Ultrasound-Assisted Thawing

**Figure 6:**
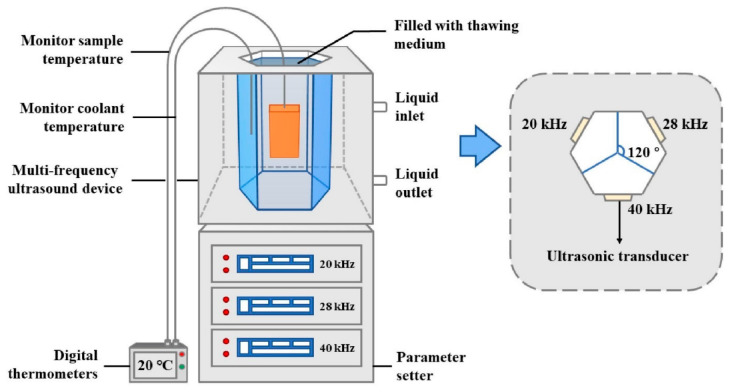
Schematic diagram of the multi-frequency ultrasonic-assisted thawing (UAT) system. The device features three ultrasonic transducers operating at distinct frequencies arranged at 120° angles. This multi-frequency configuration enhances thawing efficiency by generating complex acoustic cavitation and microstreaming effects within the liquid medium, which promotes uniform heat transfer and prevents localized thermal damage often caused by standing waves in single-frequency systems.

Ultrasound-assisted thawing (UAT) is fast, efficient and highly energy-efficient. Ultrasound is a mechanical wave with a dispersion effect, which can be transmitted in liquid media to enhance the diffusion process of substances and increase their diffusion rate. In food science applications, ultrasound is conventionally classified into two main categories: high-frequency, low-intensity diagnostic ultrasound (typically > 100 kHz to several MHz), and low-frequency, high-intensity power ultrasound (typically 20 to 100 kHz). The latter is predominantly employed in thawing processes, as its high intensity and low frequency generate significant acoustic cavitation to accelerate heat transfer [12]. When the medium has absorbed the energy from the ultrasound, the friction between the medium will cause intense high-frequency oscillations, which will then cause thermal consequences. The interface heat generation is stronger when the ultrasonic penetrates two dissimilar media. Thawed meat tissue contains both solid and liquid phases, and even after freezing there is still some liquid water present, so ultrasound has great potential for thawing frozen muscle. Studies have shown that at a power level of 400 W, the thawing times of bighead carp fillets can be significantly reduced [22]. While ultrasound improves the thawing efficiency of meat, its mechanical vibrations produce strong shear forces that also affect the protein structure of the muscle. Furthermore, after some periods of ultrasonic activity, the cavitation effect’s micro-jets and the high temperature created when the cavitation bubble bursts are rupturing the cells and starting the oxidation of the meat’s lipids, changing the fatty acid composition [85]. Hence, during sonication, attention should be made to regulate the level of ultrasonic therapy activity. According to Wu et al.’s analysis of UAT of pork fillings, UAT can shorten the period that fat oxidizes by thawing food quickly. At the same time, the weak destructive effect of 175 W/L on muscle fiber tissue reduced the release of oxidation factors. Under this ultrasonic intensity, lipid oxidation can be inhibited, effectively controlling the release of pro-oxidants that accelerate lipid oxidation due to ice crystal damage in muscle cells during freezing storage [86]. High-frequency single-frequency ultrasound may damage muscle structure and to further preserve the quality attributes of frozen meat, Bian et al. thawed frozen fish in a multi-frequency ultrasound-assisted thawing apparatus (As Shown in Figure 6). The multi-frequency UAT apparatus consists of a hexahedral ultrasonic processing system with a separate control panel and three ultrasonic transducers. Water at 20 ± 1 °C is used as the propagation medium. The thawing process is completed when the central temperature of the sample rises to 4 ± 1 °C [87]. The time required to thaw frozen samples is greatly decreased using multi-frequency UAT. Multi-frequency UAT-treated samples show improved textural characteristics, a lower TVB-N, and a lower TBARS score.

In addition to thawing frozen meat, ultrasound can help keep track of the thawing process. To investigate the relationship between ultrasonic properties and the properties of the medium and thus achieve the goal of studying the internal structural properties of the material changes, low-energy acoustic waves can be combined with Fourier transform analysis of ultrasonic time or frequency domain characteristics [88]. This has a wide range of application prospects for thawing meat.

### 4.6. Low-Temperature High-Humidity Thawing (LHT)

Low-temperature combined with high-humidity thawing (LHT) has attracted a lot of attention. This is because it increases efficiency and maintains quality while being cost effective and highly applicable. The high humidity speeds up thawing and forms a water film on the meat surface to isolate oxygen, thus slowing down the oxidation of lipids and proteins during the thawing process. Compared to room temperature thawing, the fluctuated low-temperature combined with high-humidity thawing (FLHT) treatment of beef reduced thawing loss, protein content in the drip, cooking loss, shear force, and carbonyl content by 47.27%, 42.15%, 4.58%, 12.02%, and 30.0%, respectively. Low-field nuclear magnetic resonance (NMR) relaxation measurements showed that fluctuating low temperatures combined with high humidity thawing reduced thawing losses [89]. The best and most suitable thawing method for swimming crabs was demonstrated by Ling et al. at 2–4 °C with low temperature and high humidity. After thawing, pH and TBARS values were significantly lower than after conventional thawing and close to those of fresh samples [24]. Another similar study found that FLHT effectively reduced protein aggregation by reducing turbidity and maintained the relative stability of the MPs solution and that FLHT significantly inhibited the reduction in hardness, chewiness, elasticity and cohesion of meat products by protecting the microstructure (*p* < 0.05) [25]. Overall, the low temperature and high humidity thawing technique is less costly than other new thawing techniques and is more easily applicable.

### 4.7. Vacuum Thawing

In a vacuum, the boiling point of water is lowered. When water boils in a vacuum chamber, the water vapor formed will condense on the surface of frozen food at a lower temperature. When the steam condenses, it releases latent heat, which is absorbed by the frozen food and raises the temperature of the frozen food [27]. This process is referred to as vacuum thawing. Due to the high-humidity environment maintained within the vacuum chamber, drip loss is significantly reduced. Furthermore, the extremely low oxygen concentration inherent to this environment effectively prevents the oxidative spoilage of muscle foods. Chen et al. (2020) thawed pork using a vacuum thawing technique [26]. There was no significant difference between the texture parameters of defrosted pork and that of raw pork. The thawing loss was relatively small, and the energy consumption was lower than that of defrosted pork.

One of the major problems with vacuum thawing is the loss of drip. Although ice sublimation is an endothermic process that removes heat, the primary driving force for vacuum thawing is the condensation of water vapor on the surface of the frozen food. When the steam condenses, it releases latent heat, which is absorbed by the frozen food and raises its temperature. As a result, an internal porous structure is formed and water is lost [90]. So vacuum thawing requires a combination of other technologies to make up for its shortcomings. Ultrasound-assisted vacuum thawing preserves the thermal stability of actin and maintains a more stable tertiary protein structure. Furthermore, Vacuum Steam Thawing Technology (VSTT) features a relatively low ambient temperature and an anoxic environment, which effectively prevents the oxidative degradation of frozen muscle foods. The mechanism of VSTT relies on the latent heat of water vapor condensation to defrost frozen products under vacuum conditions and is characterized by low energy consumption [27]. Based on the VSTT, the researchers proposed the Vacuum sublimation rehydration thawing (VSRT), which can be divided into two stages: “vacuum sublimation” and “rehydration heating”. In the first stage, the ice crystals of the frozen meat are sublimated to water vapor and expelled under certain vacuum conditions. The heating plate can continuously provide heat for the sublimation process and therefore the sublimation process can be controlled by adjusting the temperature of the heating plate. On the cryogenic surface of the frozen product and its interior pore channels, the water vapor then condenses and releases heat to achieve thawing [91]. VSRT has the benefit of hastening the thawing process by increasing the volume and rate of condensation during rehydration thanks to the pores and channels generated during sublimation. In the case of frozen goods, the loss of water during sublimation is made up for by the absorption of water during the subsequent rehydrating process, and the textural characteristics are unaffected by the earlier drop in water content. Additionally, water vapor rather than liquid water permeates the surface and inside of the frozen product during the VSRT rehydration process. Therefore, there is no microbiological risk associated with the VSRT method. These operational parameters are highly dependent on the specific product characteristics and equipment design. For instance, in a specific experimental setup evaluating frozen pork, Chen et al. (2020) reported that the VSRT system achieved optimal performance at a sublimation time of 19 min, a heating plate temperature of 26 °C, a rehydration water volume of 1634 mL, and a rehydration water temperature of 29 °C [26]. It must be strongly emphasized that these are strictly product- and equipment-specific findings from a single laboratory study. They do not characterize the technology’s general performance envelope and should not be extrapolated without further rigorous validation under different operational configurations, industrial scales, or distinct product geometries.

## 5. Critical Synthesis and Future Perspectives

Moving beyond individual technology descriptions, a critical synthesis of the current literature reveals several inherent compromises and conflicting findings that must be navigated for future industrial application. For instance, a persistent conflict exists between thawing rapidity and temperature uniformity. While high energy electromagnetic fields (especially microwaves) dramatically reduce thawing times, they frequently suffer from localized thermal runaway, leading to partial cooking of the muscle surface while the core remains frozen [92]. Conversely, low temperature combined with high humidity (LHT) thawing offers excellent temperature uniformity and quality preservation but struggles to meet the rapid turnover demands of large volume meat processing. Similarly, conflicting findings are observed in High Voltage Electric Field (HVEF) treatments [93,94]. While some studies praise its ability to inhibit microbial growth, others report that the inevitable generation of ozone severely accelerates lipid oxidation, particularly in aquatic muscle foods rich in polyunsaturated fatty acids. Identifying these contradictions highlights significant knowledge gaps in the current research landscape. The meat industry exhibits a strong interest in adopting these innovations to minimize drip loss and accelerate processing times; however, the most glaring gap is the overwhelming reliance on small, regularly shaped, laboratory scale samples (for example, fillets weighing 50 to 200 g) [95]. There is a critical lack of pilot scale data regarding how these technologies perform on massive, irregularly shaped commercial meat blocks. Furthermore, current literature severely lacks comprehensive Life Cycle Assessments detailing the true energy demand, carbon footprint, and economic feasibility (Return on Investment) of installing these novel systems in existing cold chain infrastructures. Widespread industrial implementation, which the authors project could be achieved within the next five to ten years given the current pace of pilot-scale developments, faces primary limitations such as high initial capital investments for specialized equipment and the technical difficulty of ensuring uniform heat distribution. To address these limitations, future efforts must focus on developing continuous flow thawing systems equipped with smart temperature sensors to dynamically monitor the process in real time [81], and integrating combined thawing technologies (such as microwave coupled with ultrasound) to synergistically mitigate the negative effects of single methods.

From an operational and economic perspective, the power requirements and resource utilization of these emerging technologies vary significantly. Electromagnetic methods, such as microwave (MW) and radio frequency (RF) thawing, demand high peak power and substantial electrical resources to operate. However, this high instantaneous energy demand is often offset by the drastically reduced thawing time (such as shrinking from hours down to minutes), which decreases the overall energy consumed by long term ambient temperature control. Conversely, low temperature combined with high humidity (LHT) thawing has lower instantaneous power requirements but necessitates the continuous operation of humidification and cooling systems over an extended period [96]. Furthermore, when these methods are applied in combination (for example, MW coupled with ultrasound), the total power requirements and initial capital investments inherently increase. Nevertheless, the economic efficiency of combined technologies must be evaluated holistically. The synergistic effects of combined methods often resolve the limitations of single technologies. A notable example is mitigating the localized thermal runaway of MW through the uniform acoustic cavitation of ultrasound. This combination effectively prevents the loss of premium product quality and minimizes drip loss, directly translating to higher sellable weight and better economic returns. Therefore, despite the higher initial resource utilization, combined thawing technologies can offer a superior long-term cost to benefit ratio for industrial meat processing. From an industrial readiness perspective, not all technologies are equally viable at present. To provide actionable guidance for the meat industry, it is crucial to identify specific directions and technological combinations that represent the most promising avenues for immediate industrial investment. For large-scale batch processing, radio frequency (RF) thawing stands out as the most promising standalone method. RF systems offer unparalleled volumetric heating and deep penetration, making them highly suitable for rapidly and uniformly thawing large, irregularly shaped frozen commercial blocks without the severe edge-overheating typically associated with microwaves. Conversely, for enterprises prioritizing the preservation of meat microstructure and minimizing drip loss over absolute thawing speed, low-temperature combined with high-humidity (LHT) thawing provides the highest economic efficiency and lowest technical barrier for equipment modification. Furthermore, to achieve an optimal balance between processing speed and product quality, the integration of microwave (MW) coupled with ultrasound-assisted thawing (UAT) represents the most scientifically robust hybrid approach. The synergistic effect of this combination effectively resolves the limitations of MW alone by mitigating localized thermal runaway through the uniform acoustic cavitation of ultrasound. This directly prevents the loss of premium product quality and minimizes drip loss, translating to higher sellable weight and better economic returns. In contrast, technologies such as standalone ultrasound and vacuum thawing currently face severe scalability bottlenecks, such as acoustic wave attenuation in large solid blocks and the high cost of maintaining massive vacuum chambers, which keep them primarily at the laboratory scale. Ultimately, the future direction of the meat industry lies in adopting these tailored or combined technological strategies, supported by AI-driven dynamic monitoring systems, to maximize both product quality and processing profitability.

As established in the Section 2 framework, a robust synthesis must evaluate these methods across four core criteria: operational efficiency, product quality, microbiological safety, and industrial viability. While the specific trade-offs of RF, MW, UAT, and LHT have been synthesized in detail above, it is imperative to contextualize Ohmic and Vacuum thawing within this same strict framework. Ohmic thawing excels in both operational efficiency and microbiological safety due to its extremely rapid, uniform volumetric heating; however, its industrial viability is currently moderated by potential toxicological risks at the electrode interface and its unsuitability for matrices with highly variable conductivities. Conversely, Vacuum thawing provides exceptional product quality and safety by establishing a hypoxic environment that halts lipid oxidation, yet its industrial scalability remains inferior due to the high capital maintenance of massive vacuum chambers and slower operational rates. To prevent narrative redundancy while ensuring that all seven technologies are systematically and comprehensively evaluated against the four framework criteria, these synthesized findings are consolidated in the definitive qualitative matrix below. To provide a clear, functional synthesis of these methods, Table 2 presents a qualitative matrix comparing all seven novel technologies against conventional methods. This side-by-side evaluation assesses critical dimensions including thawing rate, drip loss control, lipid oxidation risk, protein integrity, microbial safety profile, industrial readiness, and relative energy demand.

From a systemic food safety perspective, thawing represents a Critical Control Point (CCP) within the Hazard Analysis and Critical Control Point (HACCP) framework. According to regulatory guidance from the USDA Food Safety and Inspection Service (FSIS) and the European Food Safety Authority (EFSA), the time-temperature profile during thawing is the primary determinant of pathogen outgrowth [97,98]. Conventional prolonged air or water thawing often exposes the surface of muscle foods to ‘danger zone’ temperatures for extended periods, significantly elevating the risk of microbial proliferation [97]. In contrast, rapid volumetric and electrical heating technologies (such as MW, RF, and Ohmic thawing) drastically compress the processing duration, effectively minimizing the time the product spends in temperature ranges conducive to pathogen growth. Furthermore, certain emerging technologies offer dual-action safety benefits; for instance, ultrasound-assisted thawing (via acoustic cavitation) and specific HVEF configurations (via targeted reactive species generation) possess inherent non-thermal bactericidal properties [93]. For industrial adoption, meat processing enterprises must strictly align these distinct technological risk profiles with international regulatory mandates to ensure overarching microbiological safety.

**Table 2:** Structured cross-technology comparison of emerging thawing methods based on operational efficiency, quality preservation, and industrial viability.

Thawing Technology	Thawing Rate	Drip Loss Control	Lipid Oxidation Risk	Protein Integrity	Microbial Safety Profile	Industrial Readiness	Relative Energy Demand	Reference
High-Voltage Electric Field (HVEF)	Moderate	Superior	High (*if ozone is generated*)	Moderate	Superior	Inferior	High	[6,14,15,62,63,64]
Ohmic	Superior	Superior	Low	Superior	Superior	Moderate	High	[10,16,67,68,69]
Microwave (MW)	Superior	Inferior (*due to unevenness*)	Moderate	Inferior (*localized denaturation*)	Superior	Superior	High	[18,19,74,75,76,92]
Radiofrequency (RF)	Superior	Superior	Low	Superior	Superior	Superior	High	[19,20,20,21,82,83,84]
Ultrasound-Assisted (UAT)	Superior	Moderate	Moderate	Moderate (*mechanical shear*)	Moderate	Inferior	Moderate	[12,22,85,86,87]
Low-temp High-humidity (LHT)	Inferior (*Slow*)	Superior	Low	Superior	Moderate	Superior	Moderate	[24,25,89,96]
Vacuum	Moderate	Inferior (*sublimation loss*)	Low	Superior	Superior	Inferior	Low	[26,27,90,91]

## 6. Conclusions

Freezing is a fundamental preservation method for global meat and seafood trade, yet the subsequent thawing process remains a highly complex challenge that traditionally inflicts irreversible damage on the physicochemical, nutritional, and structural quality of muscle foods. This comprehensive review highlights that rather than relying on a single universal method, industrial technology selection must be driven by specific processing goals. For instance, rapid volumetric heating (e.g., Microwave, Radiofrequency, Ohmic) offers significant actionable advantages in minimizing the time products spend in the microbiological “danger zone” (aligning with HACCP safety frameworks), whereas emerging non-thermal methods (e.g., HVEF, Ultrasound, Vacuum, LHT) excel in mitigating drip loss and preserving microstructural integrity, despite their distinct operational limitations.

Currently, at this moment in the field, the application of novel thawing technologies remains largely bottlenecked at the laboratory or pilot scale. As synthesized in this review, there is a major problem with applying these methods independently. Single physical fields inherently possess unavoidable physical trade-offs—such as the thermal runaway in pure microwave thawing, the limited penetration depth of high-voltage electric fields, or the acoustic attenuation of ultrasound in large solid blocks. Therefore, the current state-of-the-art necessitates a definitive paradigm shift away from standalone methods towards tailored, combined thawing strategies. Looking ahead, to predict future scientific ideas and innovations in this area, the next breakthroughs will likely rely on three key pillars:

(i) Multi-Physics Synergy at an Industrial Scale: Future research will move beyond simple dual-combinations towards developing novel equipment that simultaneously couples electromagnetic, acoustic, and thermodynamic fields (e.g., RF combined with vacuum-steam) to completely neutralize the weaknesses of independent methods.
(ii) Digital Twins and AI Integration: We predict the widespread application of machine learning and digital twin models. These advanced algorithms will simulate real-time phase changes and dynamically adjust energy inputs (such as shifting frequencies or altering power levels) to perfectly accommodate the uneven moisture distribution and irregular geometry of commercial meat blocks.
(iii) Standardized Sustainability Metrics: As the industry scales these combined high-efficiency systems, future studies must prioritize standardized Life Cycle Assessments (LCA). Quantifying the exact carbon footprint and specific energy consumption will be essential to ensure these innovations align with global low-carbon economic targets. Ultimately, overcoming the existing scalability and economic barriers through these future scientific advancements will be the key to revolutionizing muscle food thawing practices, thereby enhancing the overall profitability, safety, and sustainability of the global food industry.

## Figures and Tables

**Table 1:** Overview of novel thawing technologies applied to various muscle foods: processing conditions, key outcomes, and limitations.

Thawing Technology	Food Material	Processing Condition	Conclusions	Reference
High-Voltage Electric Field (HVEF)	Beef	The needle electrode numbers of 48, 16 and 8	The thawing time of frozen samples increased significantly with a decrease in the number of needle electrodes; the shortest thawing time was achieved at a needle electrode count of 48.	[13]
	Chicken	Electric field strengths of 1.5, 2.25 and 3 kV/cm	An appropriate increase in voltage to reduce the electrode gap can shorten the thawing time and can reduce thawing losses and drip losses.	[14]
	Tuna fish	Voltages of 7.5, 10.5 and 6.10 kV; 5, 13.5 and 7.5 kV; electrode gaps of 10, 5.14 and 3 cm, respectively	Increasing the voltage and reducing the electrode gap resulted in a significant increase in TBA values; fish blocks oxidise faster at High-Voltage electric field strengths than at low electric field strengths.	[15]
Ohmic	Beef	Electric field strengths of 10, 13 and 16 V/cm	Uniform thawing; low potential for microbial contamination	[16]
	Tuna fish	Electric field strengths of 40, 50, 60 V/cm	Increase in thawing rate and protein solubility with increasing voltage	[10]
	Tuna fish	Current direction in parallel or series Electrolytic frequency 50 Hz to 20 kHz	electrical conductivity (EC) values increased with increased temperature and moisture content of the muscle.	[17]
Microwave (MW)	Pork	Microwave combined with ultrasound, 35 °C water immersion, 4 °C refrigeration, air convection, running water thawing	There were no significant differences between the six thawing treatments in terms of pH, TVB-N or conductivity. The microwave combination thawed evenly, with the best results in combination with ultrasound.	[18]
	Red seabream fish	The power is set to 300 W and the frequency to 2450 MHZ.	Low degree of denaturation of muscle proteins	[8]
	Prawn meat	Operating at 915 MHz; power of 500 W and 1 kW	Uniform internal temperature distribution, but localized surface overheating	[19]
Radiofrequency (RF)	Beef	Electrode gap from 9 cm to 19 cm; Conveyor speeds from 1 m/h to 60 m/h; Parallel electrode plate 6 kW, 27.12 MHz	Best uniformity of RF thawing at 0 min ± static conditions and at a speed of 2 m/h moving on a conveyor belt	[20]
	Prawn meat	Operating frequency 27.12 MHz; maximum power 2 kW; electrode width and length 43 cm and 100 cm respectively; maximum gap setting 160 mm	Uniform temperature distribution, no local surface overheating	[19]
	Grass carp mince	Input power 600 W; electrode gap 7 cm; voltage 1000 to 1500 V	Radiofrequency penetrates to a great depth and is suitable for thawing frozen fish	[21]
Ultrasound-Assisted (UAT)	Fish	Ultrasonic intensity of 0.135 W/mL	UAT thawing time is short, colour and pH are maintained and lipid oxidation of the sample is inhibited	[22]
	Pork	The ultrasound processing period was 20 s/T with a power density of 90,120, or 150 W/L, respectively, at 4 ± 0.1 °C	Thawing time decreases with increasing power density; reduces the flow and loss of fixed and free water in PGP samples.	[12]
	Fish	Ultrasonic frequencies in single, dual and multi-frequencies	The increase in the number of ultrasonic frequencies enhances the cavitation effect. Multi-frequency processed samples retain better quality properties	[23]
Low-temp High-humidity (LHT)	Swimming crabs	Thawing ambient temperatures of −1 °C to 1 °C, 2–4 °C, 5–7 °C and 8–10 °C; relative humidity d > 95%	Thawing at a low temperature and high humidity of 2–4 °C is the best and most suitable method of thawing. The muscle mass and functional properties of myofibrillar proteins are well maintained.	[24]
	Pork	Relative humidity of 90% and temperature at 3 °C for 6 h, then reaching 3 °C and holding for 2 h	Reduces protein aggregation and maintains relative stability; effectively improves its elastic gel network and weaving properties	[25]
Vacuum	Pork	The volume of rehydration water is controlled at 360 mL and the temperature of the rehydration water is 23 °C	The energy consumption is relatively low and the thawing rate is 77% higher than natural air thawing.	[26]
	Pork	Initial sublimation in the range of 0 to 15%	12% sublimation dehydration for the fastest thawing rate	[27]

## Data Availability

No new data were created or analyzed in this study. Data sharing is not applicable to this article.

## References

[1] Hou R., Li G., Chen P. (2023). Development Strategy Analysis of Cold Chain Logistics of Fresh Agricultural Products under the Background of Low-carbon Economy. SHS Web Conf..

[2] Jiang H.Q., Li D.N., Shao D.C., Song Z.Q., Si X., Li B. (2026). Comparative evaluation of emerging and traditional thawing technologies on quality attributes, water migration, microstructure, and volatile compounds of frozen strawberries. Food Chem..

[3] Pu A.F., Zhang Y.L., Liu G.S. (2025). Innovative magnetic field assisted freezing technology in muscle foods: Principles, applications and future prospects. Trends Food Sci. Technol..

[4] Sarah M., Madinah I. (2024). Analysis of drip loss and thermal destruction rate of tuna fillets during the low-temperature preservation period. Appl. Food Res..

[5] Jiang Q.Y., Zhang M., Mujumdar A.S. (2023). Application of physical field-assisted freezing and thawing to mitigate damage to frozen food. J. Sci. Food Agric..

[6] Jiang L., Liu D.H., Yu S.F., Zhou J.W. (2025). Advancements and perspectives of novel freezing and thawing technologies effects on meat: A review. Food Res. Int..

[7] Zhang Y., Ding C. (2020). The Study of Thawing Characteristics and Mechanism of Frozen Beef in High Voltage Electric Field. IEEE Access.

[8] Cai L., Cao M., Cao A., Regenstein J., Li J., Guan R. (2018). Ultrasound or microwave vacuum thawing of red seabream (*Pagrus major*) fillets. Ultrason. Sonochem..

[9] Cai L., Wan J., Li X., Li J. (2020). Effects of different thawing methods on physicochemical properties and structure of largemouth bass (*Micropterus salmoides*). J. Food Sci..

[10] Keshani M., Zamindar N., Hajian R. (2022). Physicochemical properties of frozen tuna fish as affected by immersion ohmic thawing and conventional thawing. Food Sci. Technol. Int..

[11] Peng Z., Zhu M., Zhang J., Zhao S., He H., Kang Z., Ma H., Xu B. (2021). Physicochemical and structural changes in myofibrillar proteins from porcine longissimus dorsi subjected to microwave combined with air convection thawing treatment. Food Chem..

[12] Wu Z., Ma W., Xue S.J., Zhou A., Liu Q., Hui A., Shen Y., Zhang W., Shi J. (2022). Ultrasound-assisted immersion thawing of prepared ground pork: Effects on thawing time, product quality, water distribution and microstructure. Lwt-Food Sci. Technol..

[13] Amiri A., Mousakhani-Ganjeh A., Shafiekhani S., Mandal R., Singh A.P., Kenari R.E. (2019). Effect of high voltage electrostatic field thawing on the functional and physicochemical properties of myofibrillar proteins. Innov. Food Sci. Emerg. Technol..

[14] Rahbari M., Hamdami N., Mirzaei H., Jafari S.M., Kashaninejad M., Khomeiri M. (2018). Effects of high voltage electric field thawing on the characteristics of chicken breast protein. J. Food Eng..

[15] Mousakhani-Ganjeh A., Hamdami N., Soltanizadeh N. (2016). Effect of high voltage thawing on the lipid oxidation of frozen tuna fish (*Thunnus albacares*). Innov. Food Sci. Emerg. Technol..

[16] Cevik M., Icier F. (2021). Numerical simulation of temperature histories of frozen minced meat having different fat contents during ohmic thawing. Int. J. Therm. Sci..

[17] Liu L., Llave Y., Jin Y., Zheng D.Y., Fukuoka M., Sakai N. (2017). Electrical conductivity and ohmic thawing of frozen tuna at high frequencies. J. Food Eng..

[18] Zhu M.M., Peng Z.Y., Lu S., He H.J., Kang Z.L., Ma H.J., Zhao S.M., Wang Z.R. (2020). Physicochemical Properties and Protein Denaturation of Pork Longissimus Dorsi Muscle Subjected to Six Microwave-Based Thawing Methods. Foods.

[19] Palazoglu T.K., Miran W. (2017). Experimental comparison of microwave and radio frequency tempering of frozen block of shrimp. Innov. Food Sci. Emerg. Technol..

[20] Bedane T.F., Chen L., Marra F., Wang S. (2017). Experimental study of radio frequency (RF) thawing of foods with movement on conveyor belt. J. Food Eng..

[21] Chen Y., He J., Li F., Tang J., Jiao Y. (2021). Model food development for tuna (*Thunnus Obesus*) in radio frequency and microwave tempering using grass carp mince. J. Food Eng..

[22] Li D., Zhao H., Muhammad A.I., Song L., Guo M., Liu D. (2020). The comparison of ultrasound-assisted thawing, air thawing and water immersion thawing on the quality of slow/fast freezing bighead carp (*Aristichthys nobilis*) fillets. Food Chem..

[23] Ma X., Mei J., Xie J. (2021). Effects of multi-frequency ultrasound on the freezing rates, quality properties and structural characteristics of cultured large yellow croaker (*Larimichthys crocea*). Ultrason. Sonochem..

[24] Ling J., Xuan X., Xu Z., Ding T., Lin X., Cui Y., Liu D. (2021). Low-temperature combined with high-humidity thawing improves the water-holding capacity and biochemical properties of *Portunus trituberculatus* protein. Food Qual. Saf..

[25] Zhu M., Li H., Xing Y., Ma C., Peng Z., Jiao L., Kang Z., Zhao S., Ma H. (2023). Understanding the influence of fluctuated low-temperature combined with high-humidity thawing on gelling properties of pork myofibrillar proteins. Food Chem..

[26] Chen S., Wu W., Yang Y., Wang H., Zhang H. (2020). Experimental study of a novel vacuum sublimation-rehydration thawing for frozen pork. Int. J. Refrig..

[27] Kopec A., Mierzejewska S., Bac A., Diakun J., Piepiorka-Stepuk J. (2022). Modification of the vacuum-steam thawing method of meat by using the initial stage of sublimation dehydration. Sci. Rep..

[28] Qiu L.Q., Zhang M., Chitrakar B., Bhandari B. (2020). Application of power ultrasound in freezing and thawing Processes: Effect on process efficiency and product quality. Ultrason. Sonochem..

[29] Llave Y., Erdogdu F. (2020). Radio frequency processing and recent advances on thawing and tempering of frozen food products. Crit. Rev. Food Sci. Nutr..

[30] Fernández-Martín F., Otero L., Solas M.T., Sanz P.D. (2000). Protein Denaturation and Structural Damage During High-Pressure-Shift Freezing of Porcine and Bovine Muscle. J. Food Sci..

[31] Sigurgisladottir S., Ingvarsdottir H., Torrissen O.J., Cardinal M., Hafsteinsson H. (2000). Effects of freezing/thawing on the microstructure and the texture of smoked Atlantic salmon (*Salmo salar*). Food Res. Int..

[32] Deng X., Huang H., Huang S., Han L., Zhang D. (2022). Insight into the incredible effects of microwave heating: Driving changes in the structure, properties and functions of macromolecular nutrients in novel food. Front. Nutr..

[33] Im C., Song S., Cheng H., Park J., Kim G.-D. (2024). Assessing Individual Muscle Characteristics to Enhance Frozen-Thawed Meat Quality. Food Sci. Anim. Resour..

[34] Park M.H., Kim M. (2024). Effects of Thawing Conditions on the Physicochemical and Microbiological Quality of Thawed Beef. Prev. Nutr. Food Sci..

[35] Shang X., Yan X., Li Q., Liu Z., Teng A. (2020). Effect of Multiple Freeze-Thaw Cycles on Myoglobin and Lipid Oxidations of Grass Carp (*Ctenopharyngodon idella*) Surimi with Different Pork Back Fat Content. Food Sci. Anim. Resour..

[36] Honikel K.O. (1998). Reference methods for the assessment of physical characteristics of meat. Meat Sci..

[37] Li X., Wei X., Wang H., Zhang C.H., Mehmood W. (2018). Relationship Between Protein Denaturation and Water Holding Capacity of Pork During Postmortem Ageing. Food Biophys..

[38] Xu C.C., Liu D.K., Guo C.X., Wu Y.Q. (2020). Effect of cooling rate and super-chilling temperature on ice crystal characteristic, cell structure, and physicochemical quality of super-chilled fresh-cut celery. Int. J. Refrig..

[39] Huff-Lonergan E., Lonergan S.M. (2005). Mechanisms of water-holding capacity of meat: The role of postmortem biochemical and structural changes. Meat Sci..

[40] Puolanne E., Halonen M. (2010). Theoretical aspects of water-holding in meat. Meat Sci..

[41] Li X., Ha M., Warner R.D., Dunshea F.R. (2022). Meta-analysis of the relationship between collagen characteristics and meat tenderness. Meat Sci..

[42] Daszkiewicz T., Purwin C., Kubiak D., Fijalkowska M., Kozlowska E., Antoszkiewicz Z. (2018). Changes in the quality of meat (Longissimus thoracis et lumborum) from Kamieniec lambs during long-term freezer storage. Anim. Sci. J..

[43] Zhang M., Li F., Diao X., Kong B., Xia X. (2017). Moisture migration, microstructure damage and protein structure changes in porcine longissimus muscle as influenced by multiple freeze-thaw cycles. Meat Sci..

[44] Chan J.T.Y., Omana D.A., Betti M. (2011). Effect of ultimate pH and freezing on the biochemical properties of proteins in turkey breast meat. Food Chem..

[45] Jiang Q., Jia R., Nakazawa N., Hu Y., Osako K., Okazaki E. (2019). Changes in protein properties and tissue histology of tuna meat as affected by salting and subsequent freezing. Food Chem..

[46] Lan W., Zhao Y., Gong T., Mei J., Xie J. (2021). Effects of different thawing methods on the physicochemical changes, water migration and protein characteristic of frozen pompano (*Trachinotus ovatus*). J. Food Biochem..

[47] Kehoe J.J., Brodkorb A., Molle D., Yokoyama E., Famelart M.H., Bouhallab S., Morris E.R., Croguennec T. (2007). Determination of exposed sulfhydryl groups in heated beta-lactoglobulin A using IAEDANS and mass spectrometry. J. Agric. Food Chem..

[48] Leygonie C., Britz T.J., Hoffman L.C. (2012). Meat quality comparison between fresh and frozen/thawed ostrich *M. iliofibularis*. Meat Sci..

[49] Xiong Z.Y., Zhou M.Q., Chen X., Xiao S. (2026). Allicin-mediated protection of myofibrillar proteins in refrigerated mandarin fish (*Siniperca chuatsi*): Molecular interactions with endogenous proteases and inhibition of protein degradation. Food Chem. X.

[50] Silva F.A.P., Estevez M., Ferreira V.C.S., Silva S.A., Lemos L.T.M., Ida E.I., Shimokomaki M., Madruga M.S. (2018). Protein and lipid oxidations in jerky chicken and consequences on sensory quality. Lwt Food Sci. Technol..

[51] Chen Q., Xie Y., Xi J., Guo Y., Qian H., Cheng Y., Chen Y., Yao W. (2018). Characterization of lipid oxidation process of beef during repeated freeze-thaw by electron spin resonance technology and Raman spectroscopy. Food Chem..

[52] Diao H., Lin S., Li D., Li S., Feng Q., Sun N. (2022). Control on moisture distribution and protein changes of Antarctic krill meat by antifreeze protein during multiple freeze-thaw cycles. J. Food Sci..

[53] Wang Z., He Z., Zhang D., Chen X., Li H. (2021). Effect of multiple freeze-thaw cycles on protein and lipid oxidation in rabbit meat. Int. J. Food Sci. Technol..

[54] Marin-Penalver D., Aleman A., Montero P., Gomez-Guillen M.C. (2018). Gelling properties of hake muscle with addition of freeze-thawed and freeze-dried soy phosphatidylcholine liposomes protected with trehalose. Lwt Food Sci. Technol..

[55] Wu B., Qiu C., Guo Y., Zhang C., Guo X., Bouhile Y., Ma H. (2022). Ultrasonic-assisted flowing water thawing of frozen beef with different frequency modes: Effects on thawing efficiency, quality characteristics and microstructure. Food Res. Int..

[56] Ali S., Khan M.A., Rajput N., Naeem M., Zhang W., Li C.B., Zhou G. (2022). Desmin as molecular chaperone for myofibrillar degradation during freeze-thaw cycles. Food Chem..

[57] Cheng H., Song S., Kim G.D. (2021). Frozen/thawed meat quality associated with muscle fiber characteristics of porcine longissimus thoracis et lumborum, psoas major, semimembranosus, and semitendinosus muscles. Sci. Rep..

[58] Hsieh Y.P., Cornforth D.P., Pearson A.M., Hooper G.R. (1980). Ultrastructural changes in pre- and post-rigor beef muscle caused by conventional and microwave cookery. Meat Sci..

[59] Kong D., Han R., Yuan M., Xi Q., Du Q., Li P., Yang Y., Rahman S.M.E., Wang J. (2023). Slightly acidic electrolyzed water as a novel thawing media combined with ultrasound for improving thawed mutton quality, nutrients and microstructure. Food Chem. X.

[60] Zhou P.C., Xie J. (2021). Effect of different thawing methods on the quality of mackerel (*Pneumatophorus japonicus*). Food Sci. Biotechnol..

[61] Cevik M., Icier F. (2020). Characterization of viscoelastic properties of minced beef meat thawed by ohmic and conventional methods. Food Sci. Technol. Int..

[62] Wei Y., Chen F., Yang H., Kang K. (2026). Effects of ultrasound-assisted freezing and high-voltage electric field thawing on quality of precooked duck meat. Front. Nutr..

[63] Dalvi-Isfahan M., Hamdami N., Le-Bail A., Xanthakis E. (2016). The principles of high voltage electric field and its application in food processing: A review. Food Res. Int..

[64] Park J.S., Sung B.J., Yoon K.S., Jeong C.S. (2016). The bactericidal effect of an ionizer under low concentration of ozone. BMC Microbiol..

[65] Sakr M., Liu S. (2014). A comprehensive review on applications of ohmic heating (OH). Renew. Sustain. Energy Rev..

[66] Doner D., Cokgezme O.F., Cevik M., Engin M., Icier F. (2020). Thermal Image Processing Technique for Determination of Temperature Distributions of Minced Beef Thawed by Ohmic and Conventional Methods. Food Bioprocess Technol..

[67] Kamonpatana P., Sastry S.K. (2022). Electrical conductivity of foods and food components: The influence of formulation processes. J. Food Process Eng..

[68] Luzuriaga D.A., Balaban M.O. (1996). Electrical Conductivity of Frozen Shrimp and Flounder at Different Temperatures and Voltage Levels. J. Aquat. Food Prod. Technol..

[69] Icier F., Sengün I., Turp G.Y., Kendirci P., Arserim E.H. Changes of some characteristics of cylindrical beef meatball cooked in continuous type ohmic cooking system. Proceedings of the International Conference on Bio & Food Electrothecnologies-BFE.

[70] Farag K.W., Lyng J.G., Morgan D.J., Cronin D.A. (2008). Dielectric and thermophysical properties of different beef meat blends over a temperature range of −18 to +10 °C. Meat Sci..

[71] Watanabe T., Ando Y. (2021). Evaluation of heating uniformity and quality attributes during vacuum microwave thawing of frozen apples. Lwt-Food Sci. Technol..

[72] Gabriel C., Gabriel S., Grant E.H., Halstead B.S.J., Mingos D.M.P. (1998). Dielectric parameters relevant to microwave dielectric heating. Chem. Soc. Rev..

[73] Zhu H., Shu W., Xu C., Yang Y., Huang K., Ye J. (2022). Novel electromagnetic-black-hole-based high-efficiency single-mode microwave liquid-phase food heating system. Innov. Food Sci. Emerg. Technol..

[74] Wang B., Du X., Kong B., Liu Q., Li F., Pan N., Xia X., Zhang D. (2020). Effect of ultrasound thawing, vacuum thawing, and microwave thawing on gelling properties of protein from porcine longissimus dorsi. Ultrason. Sonochem..

[75] Guo Y.Y., Kong B.H., Xia X.F., Yu T., Liu Q. (2014). Changes in Physicochemical and Protein Structural Properties of Common Carp (*Cyprinus carpio*) Muscle Subjected to Different Freeze-Thaw Cycles. J. Aquat. Food Prod. Technol..

[76] Yang R., Chen J. (2022). Dynamic solid-state microwave defrosting strategy with shifting frequency and adaptive power improves thawing performance. Innov. Food Sci. Emerg. Technol..

[77] Cao M., Cao A., Wang J., Cai L., Regenstein J., Ruan Y., Li X. (2018). Effect of magnetic nanoparticles plus microwave or far-infrared thawing on protein conformation changes and moisture migration of red seabream (*Pagrus Major*) fillets. Food Chem..

[78] Hu R., Zhang M., Mujumdar A.S. (2022). Application of infrared and microwave heating prior to freezing of pork: Effect on frozen meat quality. Meat Sci..

[79] Sun Q., Yuan Y., Xu B., Gao S., Zhai X., Xu F., Shi J. (2025). Innovative Technologies Reshaping Meat Industrialization: Challenges and Opportunities in the Intelligent Era. Foods.

[80] Rathnayake P.Y., Yu R., Yeo S.E., Choi Y.S., Hwangbo S., Yong H.I. (2025). Application of ultrasound to animal-based food to improve microbial safety and processing efficiency. Food Sci. Anim. Resour..

[81] Xia Q., Yan S., Huang M., Chen K., Huang J. (2026). Advances in Freezing and Thawing Meat: From Physical Principles to Artificial Intelligence. Foods.

[82] Marra F., Zhang L., Lyng J.G. (2009). Radio frequency treatment of foods: Review of recent advances. J. Food Eng..

[83] Yang H., Chen Q., Cao H., Fan D., Huang J., Zhao J., Yan B., Zhou W., Hang W., Zhang H. (2019). Radiofrequency Thawing of Frozen Minced Fish Based on the Dielectric Response Mechanism. Innov. Food Sci. Emerg. Technol..

[84] Llave Y., Terada Y., Fukuoka M., Sakai N. (2014). Dielectric properties of frozen tuna and analysis of defrosting using a radio-frequency system at low frequencies. J. Food Eng..

[85] Koerzendoerfer A. (2022). Vibrations and ultrasound in food processing—Sources of vibrations, adverse effects, and beneficial—An overview. J. Food Eng..

[86] Sun Q., Chen Q., Xia X., Kong B., Diao X. (2019). Effects of ultrasound-assisted freezing at different power levels on the structure and thermal stability of common carp (*Cyprinus carpio*) proteins. Ultrason. Sonochem..

[87] Bian C., Cheng H., Yu H., Mei J., Xie J. (2022). Effect of multi-frequency ultrasound assisted thawing on the quality of large yellow croaker (*Larimichthys crocea*). Ultrason. Sonochem..

[88] El Kadi Y.A., Moudden A., Faiz B., Maze G., Decultot D. (2013). Ultrasonic monitoring of fish thawing process optimal time of thawing and effect of freezing/thawing. Acta Sci. Pol. Technol. Aliment..

[89] Li Y., Jia W., Zhang C.H., Li X., Wang J.Z., Zhang D.Q., Mu G.F. (2014). Fluctuated Low Temperature Combined with High-Humidity Thawing to Reduce Physicochemical Quality Deterioration of Beef. Food Bioprocess Technol..

[90] Chen S.S., Wu W.D., Liu F.R., Zhang H., Yang W.F. (2022). Experimental study on the effect of heating plate (Heat source) temperature on a new vacuum sublimation—Rehydration thawing. Int. J. Refrig..

[91] Chen S.S., Wu W.D., Mao S.J., Li K., Zhang H. (2023). Optimization of a novel vacuum sublimation-rehydration thawing process. J. Food Sci..

[92] Paoletti U., Ichikawa K., Kaneko T., Iizuka A. (2024). Contribution of Electric Field Polarization to Temperature Runaway in Radio-Frequency Thawing and Design-Oriented Simulations. IEEE Access.

[93] Peng J., Liu C., Xing S., Bai K., Liu F. (2023). The application of electrostatic field technology for the preservation of perishable foods. Food Sci. Technol..

[94] Wang W., Liu L., Tian M., Sun X., Shi R., Li J., Wang D., Yang Q., Zhang D., Hou C. (2024). Synergistic Preservation of Fresh Pork: Coupling Electrostatic Field and Packaging During Controlled Freezing-Point Storage. Foods.

[95] Jiang J., Zhou F., Xian C., Shi Y., Wang X. (2021). Effects of Radio Frequency Tempering on the Texture of Frozen Tilapia Fillets. Foods.

[96] Cai L., Cao M., Regenstein J.M., Cao A. (2019). Recent advances in food thawing technologies. Compr. Rev. Food Sci. Food Saf..

[97] USDA FSIS (2013). The Big Thaw—Safe Defrosting Methods.

[98] EFSA Panel on Biological Hazards (BIOHAZ) (2014). Scientific Opinion on the public health risks related to the maintenance of the cold chain during storage and transport of meat. EFSA J..

